# Quadratus Lumborum Block for Ovarian Cystectomy Surgery in a Patient With Severe Kyphoscoliosis

**DOI:** 10.7759/cureus.51513

**Published:** 2024-01-02

**Authors:** Zhi Yuen Beh, Chuang Shin Mok, Woon Lai Lim, Hing Wa Yip, Pui San Loh, Mohd Afiq Syahmi Ramli, Siti Zawiah Omar

**Affiliations:** 1 Anaesthesiology, University of Malaya Medical Centre, Kuala Lumpur, MYS; 2 Anaesthesia, Gleneagles Hospital Kuala Lumpur, Kuala Lumpur, MYS; 3 Obstetrics and Gynaecology, University Malaya, Kuala Lumpur, MYS

**Keywords:** type 3 osteogenesis imperfecta, ultrasound guided nerve block, ovarian cystectomy, abdominal laparotomy, anesthesia and analgesia, : regional anesthesia

## Abstract

Quadratus lumborum block (QLB) has been described as a regional analgesic technique in various abdominal surgeries. We present a case report of a high-risk patient who underwent ovarian cystectomy with QLB and deep sedation after failed neuraxial anesthesia. A 29-year-old female patient with comorbidities osteogenesis imperfecta, severe kyphoscoliosis with restrictive lung disease, and cervical syringomyelia with cranio-cervical junction stenosis (C2/C3). The patient had large ovarian cysts with associated dyspnea. She accepted surgery-an open bilateral ovarian cystectomy-despite being advised that general anesthesia would be high-risk. Regional anesthetic options were limited and challenging, given her anatomy and difficulty in positioning. Neuraxial anesthesia was attempted but was unsuccessful. The patient safely underwent surgery (lower midline laparotomy) using QLB. This clinically challenging case demonstrates the feasibility of QLB as the mainstay multimodal anesthetic approach (without general and neuraxial anesthesia) for abdominal surgery under exceptional circumstances.

## Introduction

Quadratus lumborum block (QLB) is an ultrasound-guided fascial plane block where local anesthetic (LA) is injected adjacent to the quadratus lumborum muscle to anesthetize the thoracolumbar nerves, which innervate the thoracoabdominal wall [1]. Since its first description by Blanco [2,3] in 2007, QLB has evolved into a heterogenous group of techniques; lateral, posterior, anterior (transmuscular), and subcostal variations have all been described [1,4]. Due to heterogeneity in the names and anatomical descriptions of various abdominal wall blocks, a recent ASRA-ESRA Delphi consensus study proposed a standard nomenclature for QLB approaches: anterior, posterior, and lateral [5]. Different approaches to the QLB have been successfully described in various abdominal surgeries, such as caesarean sections [6,7], gynecology surgery [8], and urological surgery [9]. Most were published as case reports, with growing randomized controlled studies [10]. However, the current literature only describes the successful application of QLB for postoperative analgesia [1,4,10]. To our knowledge, no study describes using QLB as surgical anesthesia for abdominal surgery. We present a case report of a high-risk patient who underwent ovarian cystectomy with QLB and deep sedation after failed neuraxial anesthesia. 

## Case presentation

**Figure 1:**
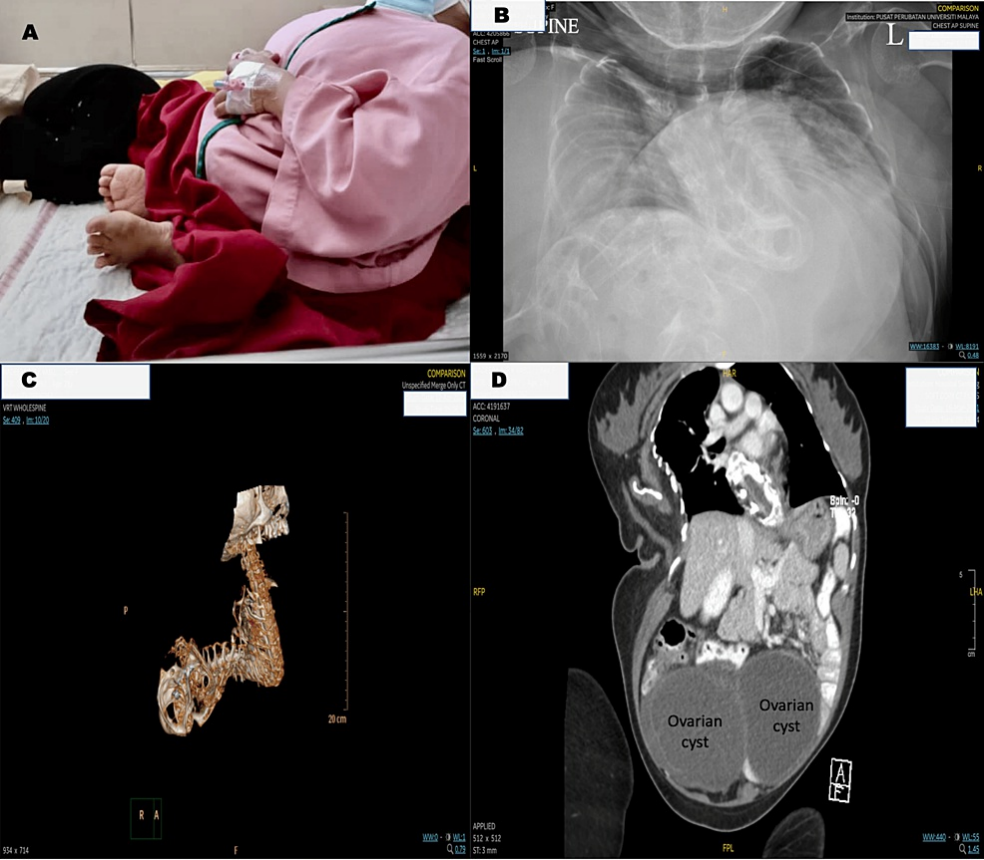
Patient's background medical problems A. The patient’s short stature and severe physical disabilities resulted from osteogenesis imperfecta. She was largely wheelchair-bound and semi-independent in the activities of daily living. B. Chest X-ray showed very poor lung reserve. Her lung function test showed severe restrictive lung disease (forced expiratory volume in 1 second, FEV1 0.36L; forced vital capacity, FVC 0.44L; FEV1/FVC 81%). C. Computed tomography (CT) of the whole spine showed severe kyphoscoliosis. Multiple fractures of varying ages were reported to be present in bilateral first ribs, the costovertebral junction from T2 to T5 levels on the right, T2 to T4 levels on the left, and the bilateral femoral neck. CT thorax, abdomen, and pelvis showed bilateral large multi-loculated ovarian cysts (right side: 12.1 x 9.3 x 10.3cm; left side: 6.8 x 9 x 7cm)

A 29-year-old, 37 kg short-stature female patient had underlying osteogenesis imperfecta (OI) Type III and severe kyphoscoliosis (computerized tomography [CT] revealed severe kyphoscoliosis and multiple fractures of varying ages in bilateral first ribs, costovertebral junction from T2 to T5 levels on the right side and T2 to T4 levels on the left side and both femoral neck) (Figure 1). She also had severe restrictive lung disease (Figure 1) with poor pulmonary reserve (spirometry: forced expiratory volume in 1 second, FEV1 0.36L; forced vital capacity, FVC 0.44L; FEV1/FVC 81%) on metered dose inhaler (MDI) Fluticasone/Salmeterol 25/250 mg 2 puffs BD and oral Montelukast 10mg ON. The patient also had cervical syringomyelia with cranio-cervical junction stenosis (C2/C3) and neurological sequelae: chronic numbness of bilateral upper limbs (medial aspect) and generalized numbness of bilateral lower limbs. She was largely wheelchair-bound and semi-independent in the activities of daily living. 

She had bilateral large multi-loculated ovarian cysts (right: 12.1 x 9.3 x 10.3cm; left: 6.8 x 9 x 7cm on abdominal CT) contributing to her progressively worsening dyspnea (Figure 1). A multidisciplinary, patient-oriented approach was taken in the decision-making process. The patient and her family were keen for surgical intervention, having understood the anesthetic difficulties involved and potential pulmonary complications that might cause significant morbidity and mortality during the perioperative period. 

We had planned for neuraxial anesthesia to be the primary anesthetic technique, with truncal blocks and multimodal analgesia as supplementary options. Given the fact that her anatomy and physiology would invariably pose unique anesthetic challenges with limited options for regional anesthesia, the possibility of abandoning the surgery had been discussed and agreed upon if surgical anesthesia could not be achieved with the proposed techniques. 

**Figure 2:**
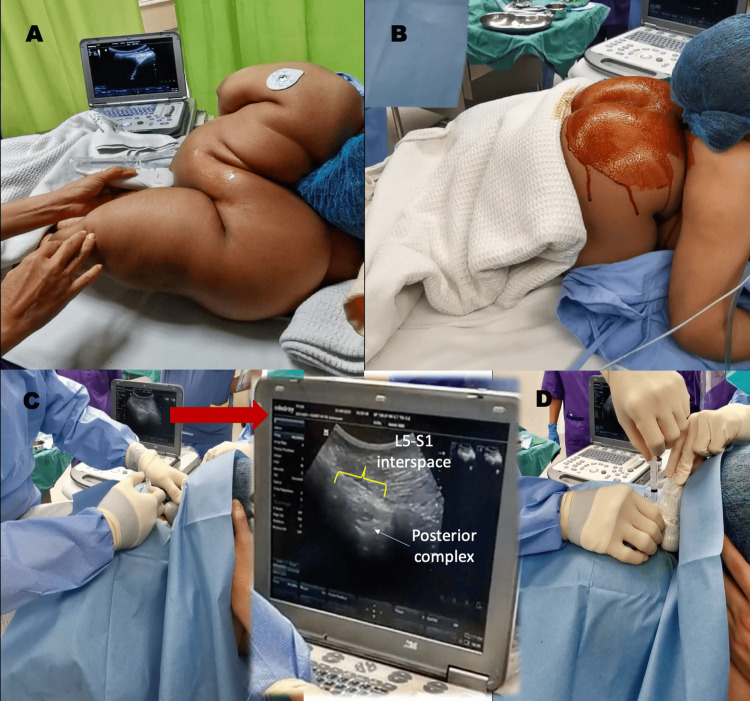
Technical challenges in performing neuraxial anesthesia A. The patient could not sit up properly, and the room for scanning plus needling in a sitting position was extremely limited. B. The patient turned to a prone kneeling position for a neuraxial technique. C. Attempting continuous spinal anesthesia under real-time ultrasound guidance using the Sprotte needle 23G (Pajunk Intralong, Geisingen, Germany). The needle was inserted in-plane caudal-to-cranial needling technique; Inset: Ultrasound of the lumbar spine (paramedian oblique sagittal view) showed L5-S1 intervertebral space and posterior complex. D. However, there was negative efflux of cerebrospinal fluid (CSF) through the spinal hub despite waiting for a minute and negative aspiration. A spinal test dose of levobupivacaine 0.5% 0.5 mL was delivered, but the patient did not experience progressive numbness or weakness of the bilateral lower limb, thus the neuraxial technique was abandoned.

**Figure 3:**
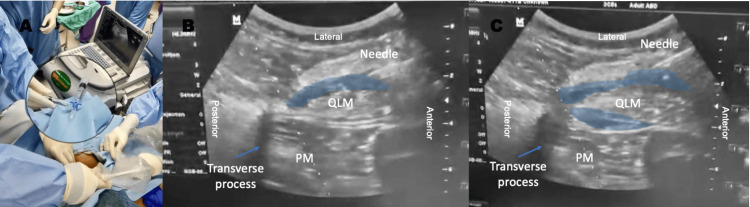
Ultrasound-guided bilateral quadratus lumborum block (QLB) and sonogram [We could not store and export the sonogram due to a faulty ultrasound storage system- pictures were taken by an amateur colleague] A. Before the local anesthetic (LA) deposit, the fascial plane was hydro-located and hydro-dissected with saline with the help of an assistant using two syringes (LA solution, 10ml, and saline, 3ml) and a three-way tap connector connecting to the Stimuplex needle (inset). B. Lateral QLB: the deposition of LA on the lateral side of the QL muscle after the needle tip penetrates the transversus abdominis muscle aponeurosis. Although LA was deposited in the lateral aspect of QL muscle, a sonogram showed LA tracking to the posterolateral aspect of QL muscle in the thoracolumbar fascia between QL muscle and paraspinal muscles. C. Anterior QLB was also performed using the same needling trajectory (anterior-to-posterior). LA was deposited in between the QL and psoas muscles. Our ultimate goal was to ensure adequate LA spread within the thoracolumbar fascial plane to improve the success of the block. Blue shade: Local anesthetic deposition; QLM: Quadratus lumborum muscle; PM: Psoas muscle

The patient could lie supine and extend both hip joints for surgical access. She was then placed on standard American Society of Anesthesiologists (ASA) monitoring, and a right radial arterial line was inserted under ultrasound guidance before the conduct of anesthesia for close blood pressure monitoring. A preliminary sonographic scout scan was performed with an ultrasound Mindray M6 (Shenzhen, P.R. China) using a low-frequency curvilinear transducer (3C5S, 1.7-6 MHz) to determine the feasibility of truncal block and neuraxial techniques (Figure 2, Video 1). We initially intended to perform continuous spinal anesthesia (CSA) as the neuraxial technique of choice. However, the patient could not sit up adequately, and there was limited room for scanning and needle insertion. Therefore, our patient was placed in a prone-kneeling position for the neuraxial technique (Figure 2, Video 1). A 23G Sprotte needle (Pajunk Intralong, Geisingen, Germany) was inserted at the L5-S1 intervertebral space under real-time ultrasound imaging (paramedian oblique sagittal view) using an in-plane approach in a caudal-cranial orientation. Although the needle was observed to have pierced the posterior complex (Figure 2), there was no efflux of cerebrospinal fluid (CSF) through the spinal needle after a waiting time of one minute. Needle aspiration was negative. A spinal test dose of levobupivacaine 0.5% 0.5 mL was delivered, but the patient did not experience progressive numbness or weakness of the bilateral lower limb; thus, the neuraxial technique was abandoned. She was then positioned supine for the truncal block. Bilateral ultrasound-guided QLB was performed using a 21G 100 mm insulated short bevel needle (Stimuplex A, BBraun, Melsungen, Germany) with local anesthetic (LA) ropivacaine 0.375% 20 ml delivered to each side (Figure 3). A lateral approach to QLB was initially performed, with LA deposited adjacent to the lateral aspect of the quadratus lumborum (QL) muscle and immediately lateral to the tapered end of the transverse abdominis muscle aponeurosis. The needle was advanced in-plane in an anterior-to-posterior direction to reach the interfascial plane. Before LA deposition, the fascial plane was hydro-located and hydro-dissected using saline. Due to unsatisfactory LA spread, we also deposited LA between the interfascial plane of the QL and psoas muscles, which were also visible in the sonogram. This was technically considered an anterior-approach QLB using a modified anterior-posterior needle trajectory. We wanted to ensure that LA spread adequately within the thoracolumbar fascia (TLF) plane to improve block success. The patient was ready for surgical incision 30 minutes after the QLB. She also received monitored sedation with intravenous multimodal analgesia: morphine 0.5mg, paracetamol 1 mg, and parecoxib 40mg were given before regional block with ketamine (titrated dose of 10mg per bolus, total 30mg) and fentanyl (titrated doses of 10 mg per bolus, total 30 mg given during actual block performance to help her cope with the discomfort). Before the surgical incision, the surgeon tested the incision site with a pinch test, and the patient did not have any reaction or surge in hemodynamic response. An additional morphine dose of 0.5mg was given, and a dexmedetomidine infusion was commenced at 0.5 mg/kg/hour (without the loading dose). The patient was kept under deep sedation (modified observer’s assessment of alertness/sedation scale 1) because she had a very low pain threshold and received oxygen supplementation delivered via a Hudson mask. The nasopharyngeal airway size of 6.0mm was inserted prophylactically, and capnography was monitored. Our patient safely underwent bilateral ovarian cystectomy, ovarian refashioning, and myomectomy for a small subserosal uterine fibroid. Her vital signs remained stable throughout the 2-hour and 10-minute surgical procedure. Surgeons initially attempted surgery via a Pfannenstiel incision, but they converted to a lower midline laparotomy due to huge ovarian cysts (Figure 3). The patient groaned when surgeons were mobilizing the cysts. Additional ketamine (10 mg) was given twice. No desaturation occurred during the perioperative period, and her arterial blood gas (ABG) under Hudson mask oxygen at 8 L/min during surgery showed neither hypoxia nor significant CO2 retention (pH 7.367, pCO2 45.3 mmHg, pO2 270 mmHg, HCO3 24.6 mmol/L). The actual sizes of the ovarian cysts were 15cm x 15cm on the left (clear fluid content) and 10cm x 10cm on the right (sebaceous content, thick-walled). The patient had a zero-pain score on the numerical rating scale (NRS) at the recovery area. She was closely monitored in the intensive care unit (ICU) for one day and received regular intravenous paracetamol and parecoxib. A repeat ABG under room air was done in the ICU, which showed normal results (pH 7.408, pCO2 36.4 mmHg, pO2 86.2 mmHg, and HCO3 23.2 mmol/L). She started feeling discomfort at the wound site with a pain score of 5 by midnight (approximately 12 hours after the surgery), and regular intravenous tramadol was started. The following day, she had more pain (average NRS 5), and subcutaneous morphine (5mg QID) was added to the multimodal analgesia regime. The patient remained stable and was transferred to the general ward on postoperative day two. 

**Video 1:** Summary of the case report We uploaded a video clip that showed the patient’s short physical stature with severe kyphoscoliosis and limited positioning capability, causing significant technical challenges at the attempts of neuraxial technique and truncal block plus deep sedation during surgery.

## Discussion

To our knowledge, this is the first published case describing QLB as surgical anesthesia for a clinically challenging case that underwent lower abdominal surgery with a multi-modal analgesia regime plus deep sedation. Currently, the literature has only described the successful use of QLB by various approaches as part of post-operative analgesia in a wide range of abdominal surgeries [1,4,6-9]. Osteogenesis imperfecta (OI) is a genetic bone disease that causes frequent fractures, which is the hallmark of its clinical presentation. Patients with OI Type III have broken bones since childhood, which often leads to severe physical disabilities by adulthood. Overall, patients with OI have shorter life expectancies than the general population, with a higher risk of death due to respiratory diseases, gastrointestinal diseases, and trauma (bone fractures) [11].

This patient unfortunately had huge bilateral ovarian cysts, which impaired her breathing and left her suffering from numerous problems and physical disabilities related to OI. She wanted surgical intervention despite knowing the high perioperative mortality risk if general anesthesia was the only choice. To avoid the risks of general anesthesia, neuraxial anesthesia is deemed the most reliable anesthetic technique to provide visceral and somatic analgesia for abdominal surgery. Dose-titration of intrathecal injectate to achieve the clinical effect through continuous spinal anesthesia is the safest approach to maintain hemodynamic stability without compromising the patient’s respiratory function and to provide effective anesthesia for abdominal surgery [12]. However, the patient’s short physical stature with severe kyphoscoliosis and limited positioning capability have caused significant technical challenges in the attempts at neuraxial technique, even under the hands of a skilled operator. Under the circumstances, we attempted truncal block bilateral ultrasound guided QLB as surgical anesthesia based on cadaveric studies [13,14] and clinical reports [15], which showed a high volume LA deposit would possibly spread to the thoracic paravertebral space along the endothoracic fascia to block the somatic nerves and thoracic sympathetic trunk of the lower thoracic levels. These explained the longer duration of the analgesic effect with visceral analgesia coverage following QLB [1,3,10]. We acknowledged that our LA dose was slightly above the maximal allowable dose per body weight of 3 mg/kg for ropivacaine. However, we desperately aimed to provide a truncal block for surgical anesthesia, and ropivacaine 0.375% was the least reliable concentration to produce the anesthetic effect in the literature [16]. A multimodal analgesic regime was concurrently administered to manage the multifaceted pain pathway and offer a synergistic effect. We had to keep the patient under deep sedation (modified observer’s assessment of alertness/sedation scale 1) because she had a very low pain threshold. Surgery proceeded after the surgeon adequately tested the incision site with a pinch test.

Based on our scout scan, QLB was feasible, although the sonoanatomy was suboptimal. We had to use a modified, combined approach with a single needling trajectory (anterior-to-posterior direction) to perform lateral and anterior QLB because of the desperate situation and the initial LA spread following the first injection; lateral QLB was unsatisfactory (Figure 3). Surgery would be abandoned if truncal block failed to provide adequate anesthetic coverage. We did not use an alternative truncal block technique, such as the erector spinae plane (ESP) block, for this case because of the patient’s anatomy and difficulty in positioning. The ESP block was also described as the truncal block for postoperative analgesia in abdominal surgeries [17]. Similar to QLB, there has been no published paper describing ESP block as surgical anesthesia for major abdominal surgery to date.

We acknowledge that surgical anesthesia for abdominal surgery solely with bilateral QLB would be highly challenging; nevertheless, QLB has proven to provide adequate analgesia. For surgical anesthesia, QLB requires a multimodal analgesia combination, and it should be reserved for those unfit for general anesthesia or neuraxial technique. It’s only worth considering if surgical intervention is deemed feasible and the benefits outweigh the risks for the patient. The visceral analgesia coverage by QLB can be challenging to predict, and the spread of QLB to thoracic PVB remains ambiguous based on several clinical evidence [1,3,4,9]. This patient had multiple neurological issues, including syringomyelia with cranio-cervical junction stenosis with chronic numbness of bilateral upper limbs and generalized numbness of bilateral lower limbs. The patient might have had neurological deficits affecting abdominal nerves that were probably not assessed pre-operatively. Therefore, we cannot claim (with certainty) that surgical anesthesia can be attributed solely to QLB, given the fact that we also used deep sedation in this case. In reality, sedation analgesia alone is inadequate for surgery involving a lower midline laparotomy. The patient gradually had increased pain severity postoperatively, which coincided with the block duration of analgesia, thus demonstrating the analgesic efficacy of QLB. 

## Conclusions

This clinically challenging case demonstrates the feasibility of QLB as the mainstay multimodal anesthetic approach (without general and neuraxial anesthesia) for abdominal surgery under exceptional circumstances.
